# Mimicking acute airway tissue damage using femtosecond laser nanosurgery in airway organoids

**DOI:** 10.3389/fcell.2023.1268621

**Published:** 2023-09-08

**Authors:** Lara Gentemann, Sören Donath, Anna E. Seidler, Lara Patyk, Manuela Buettner, Alexander Heisterkamp, Stefan Kalies

**Affiliations:** ^1^ Institute of Quantum Optics, Leibniz University Hannover, Hannover, Germany; ^2^ Lower Saxony Center for Biomedical Engineering, Implant Research and Development, Hannover, Germany; ^3^ REBIRTH Research Center for Translational Regenerative Medicine, Hannover, Germany; ^4^ Institute for Laboratory Animal Science, Hannover Medical School, Hannover, Germany; ^5^ German Center for Lung Research (DZL), Gießen, Germany

**Keywords:** airway organoids, acute lung damage, epithelial repair, laser-based nanosurgery, femtosecond laser

## Abstract

Airway organoids derived from adult murine epithelial cells represent a complex 3D *in vitro* system mimicking the airway epithelial tissue’s native cell composition and physiological properties. In combination with a precise damage induction via femtosecond laser-based nanosurgery, this model might allow for the examination of intra- and intercellular dynamics in the course of repair processes with a high spatio-temporal resolution, which can hardly be reached using *in vivo* approaches. For characterization of the organoids’ response to single or multiple-cell ablation, we first analyzed overall organoid survival and found that airway organoids were capable of efficiently repairing damage induced by femtosecond laser-based ablation of a single to ten cells within 24 h. An EdU staining assay further revealed a steady proliferative potential of airway organoid cells. Especially in the case of ablation of five cells, proliferation was enhanced within the first 4 h upon damage induction, whereas ablation of ten cells was followed by a slight decrease in proliferation within this time frame. Analyzing individual trajectories of single cells within airway organoids, we found an increased migratory behavior in cells within close proximity to the ablation site following the ablation of ten, but not five cells. Bulk RNA sequencing and subsequent enrichment analysis revealed the differential expression of sets of genes involved in the regulation of epithelial repair, distinct signaling pathway activities such as Notch signaling, as well as cell migration after laser-based ablation. Together, our findings demonstrate that organoid repair upon ablation of ten cells involves key processes by which native airway epithelial wound healing is regulated. This marks the herein presented *in vitro* damage model suitable to study repair processes following localized airway injury, thereby posing a novel approach to gain insights into the mechanisms driving epithelial repair on a single-cell level.

## 1 Introduction

Due to its physiological function, the lung is constantly exposed to external, airborne hazards such as pollutants or viruses. While this can lead to acute irritation of the lung epithelial tissue, long-term or high-dosage exposures might induce chronic inflammatory conditions (Vernooy et al., 2002; Guo et al., 2018). These pathologies are often accompanied by high rates of tissue damage, which, at a certain point, cannot be repaired by endogenous regeneration mechanisms anymore, making pulmonary diseases one of the world’s leading causes of death (Hogan et al., 2014; Barkauskas et al., 2017). While, under homeostatic conditions, the airway epithelium represents a rather quiescent tissue, it unleashes a remarkable regenerative potential with high lineage plasticity upon injury (Rao Tata and Rajagopal, 2017; Wu et al., 2022). In this context, several studies have identified Trp63^+^, Krt5^+^ basal cells as the airway epithelium’s resident stem cells serving for self-renewal and as progenitors during repair (Rock et al., 2010; Wu et al., 2022). The balance between basal cell maintenance or differentiation is regulated by the dynamic activity of different signaling pathways. Especially Notch signaling controls the fate of basal as well as their derivative luminal secretory cells during homeostasis and following injury (Tsao et al., 2009; Rock et al., 2011; Xing et al., 2012; Mori et al., 2015; Pardo-Saganta et al., 2015). While luminal Krt8^+^, Scgb1a1^+^ secretory cells can give rise to multiciliated cells under low Notch activity, the maintenance of their secretory cell state is regulated by juxtacrine Notch signals derived from basal cells (Pardo-Saganta et al., 2015). Besides their capability to further differentiate, secretory cells were shown to be able to fully restore an ablated basal cell population by dedifferentiation, thereby acting as a backup progenitor cell population (Rawlins et al., 2009; Tata et al., 2013; Wu et al., 2022). Still, on the cellular level, it has yet to be solved which events are necessary for effective repair or, *vice versa*, finally lead to the aforementioned pathologies (Barkauskas et al., 2017).

To gain a better understanding of how early endogenous regeneration and repair processes occur and how these could be controlled or regulated to ultimately enable the development of cell-based therapeutics, it is essential to study the fate of single cells upon injury. In this context, the advancement of appropriate model systems is indispensable. Organotypic models, such as stem cell-derived, self-assembled organoids, mimic the native tissue’s complex three-dimensional structure and cell heterogeneity as well as interaction, thereby resembling its biological properties to a high extent (Clevers, 2016; Barkauskas et al., 2017). For *in vitro* regeneration studies of airway epithelial tissue, several injury models have been developed, which, in a majority of cases, are based on the application of either naphthalene, sulfur dioxide, or on ionizing radiation (Borthwick et al., 2001; Hsu et al., 2014). These methods induce broad and rather unspecific damage. To study localized acute injury and reaction, the spatially selective ablation of only a limited number of cells is desirable. This would allow the identification of events specifically triggered in cells close to the injury site, potentially serving as essential factors for repair. One possible approach for such a targeted and precise damage induction in cellular systems is the employment of femtosecond laser nanosurgery (König et al., 1999; Heisterkamp et al., 2005; Vogel et al., 2005; Müller et al., 2021). Based on non-linear optical effects within biological material, this method generates a low-density plasma of free electrons which causes the bonds between molecules to break (Vogel et al., 2005). Femtosecond laser nanosurgery outperforms other manipulation techniques due to its very high spatial selectivity. As there is negligible heating and confinement of all effects to the focal volume, precise 3D application is possible without adverse effects to surrounding cells (Cheng et al., 2021; Liang and Vogel, 2022). In the context of organoids, femtosecond laser nanosurgery has previously been applied to selectively ablate single cells and study the neighboring cells’ reparative response in colonoids (Donath et al., 2022).

**FIGURE 1:**
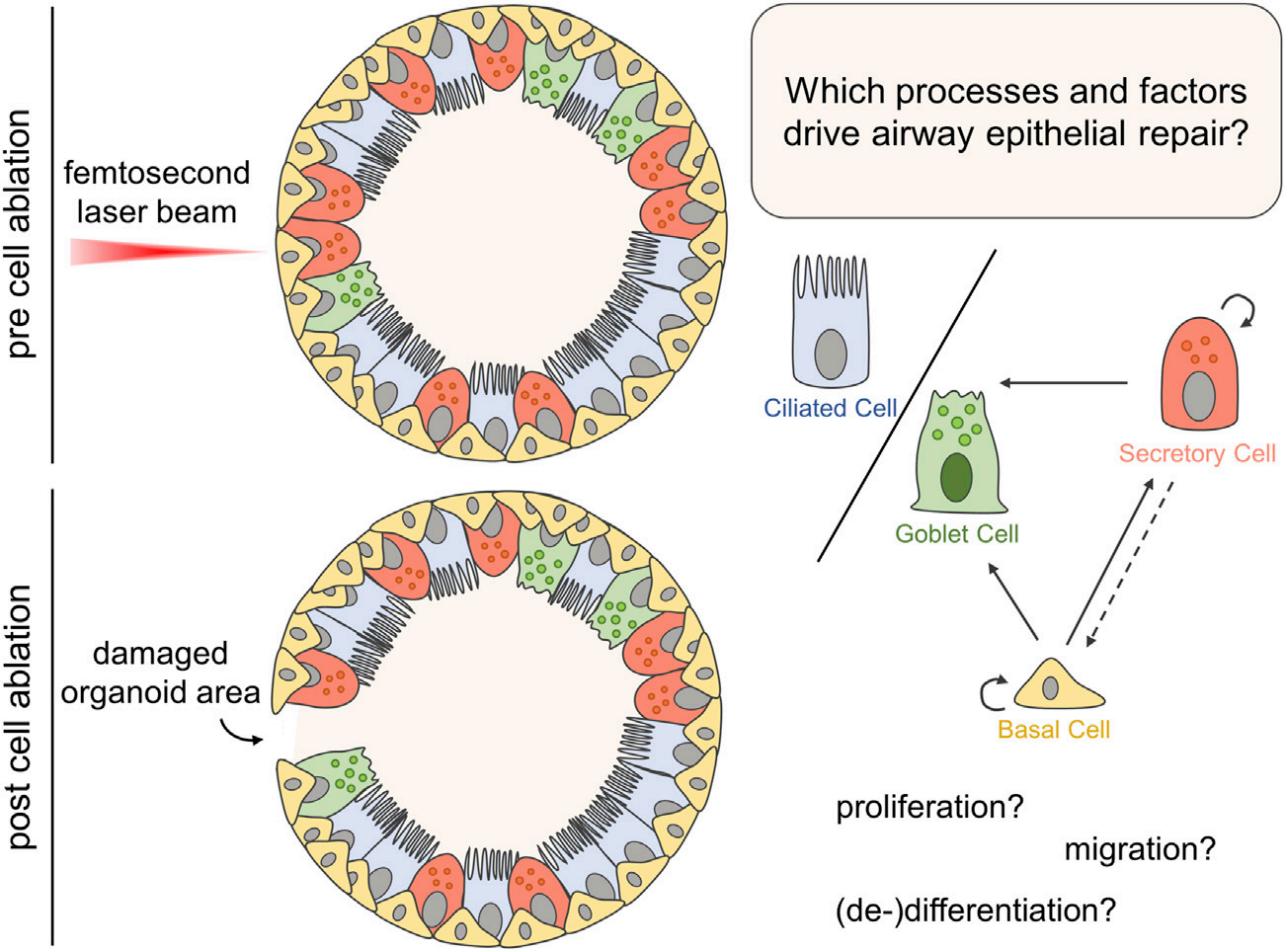
Airway organoids, mimicking the structure of proximal airway epithelial tissue mainly composed of basal as well as luminal secretory, ciliated, and goblet cells, were subjected to femtosecond laser-based nanosurgery for targeted ablation of a single or up to ten cells. Subsequently, multiphoton or confocal microscopy was employed to study the organoid’s response to the damage in terms of structural integrity, proliferation, migration and changes in gene expression to identify processes driving the epithelial repair.

Here, we apply targeted femtosecond laser-based nanosurgery in combination with state-of-the-art imaging technologies such as multiphoton or confocal microscopy to better understand epithelial repair in airway organoids (see Figure 1). The precise ablation of a single or up to ten cells enabled the investigation of the individual cells’ damage response dependent on their distance to the injured site in terms of their migratory and proliferative behavior. Moreover, we conducted RNA sequencing to support our findings on a molecular level and to identify further factors and processes functionally involved in epithelial repair after localized injury in airway organoid.

## 2 Materials and methods

### 2.1 Isolation and preparation of airway organoids

The protocol for the isolation of airway epithelial cells was based on the work of Rabata et al. (2017). The experiments complied with the German Animal Protection Act (§4, TierSchG) and were approved by the local institutional advisory committee for animal care and research and by the Lower Saxony State Office for Consumer Protection and Food Safety (file number 42500/1H).

C57BL/6J wildtype mice (both sexes, 10–14 weeks) were sacrificed by CO_2_ inhalation and subsequent cervical dislocation. The lungs were collected and kept in 1× Dulbecco’s phosphate-buffered saline (DPBS, Sigma-Aldrich, MO, United States). Under sterile conditions, the tissue was cut into small pieces which were then transferred into freshly prepared and prewarmed (37°C) digestion solution (DMEM, high glucose (Sigma-Aldrich, MO, United States) supplemented with 3 mg/mL collagenase A (Sigma-Aldrich, MO, United States), 2 mg/mL trypsin (Sigma-Aldrich, MO, United States), 5% fetal calf serum (Pan Biotech, Germany), 5 μg/mL insulin (Sigma-Aldrich, MO, United States), 1× Cellshield (Biochrom, Germany)). After incubation of 1 h at 37°C under constant agitation, the cell suspension was centrifuged for 5 min at 450 × g and 4°C, followed by two subsequent erylysis steps for 2 min and 30 s, respectively, using Red Blood Cell Lysis Buffer (155 mM NH_4_Cl, 12 mM NaHCO_3_, 0.1 mM EDTA in ddH_2_O, pH 7.4). DNA digestion was conducted in DMEM supplemented with 20 U/mL DNase I (Sigma-Aldrich, MO, United States) for 3 min at room temperature (RT). To decrease the number of stromal cells, the cell suspension was subjected to differential centrifugation three times for 10 s each, at 450 × g and 4°C. The organoid-forming epithelial cells, concentrated in the cell pellet, were further dissociated into a single-cell solution by incubation in 1× TrypLE Select (Thermo Fisher Scientific, MA, United States) for 10 min at RT and passed through a 40 μm cell strainer (VWR, PA, United States). The cells were resuspended in Cultrex Reduced Growth Factor Basement Membrane Extract (BME), Type R1 (R&D Systems, MN, United States) at a concentration of 2.5 × 10^6^ cells/mL and plated in domes of 30 µL/well in a 24-well plate (Costar^®^ Cell Culture Plate, Corning Incorporate™, NY, United States of America) preheated to 37°C. After a subsequent incubation of the plated cells for 30 min at 37°C, 5% CO_2_ for gelation of the Cultrex, organoid growth medium (DMEM, high glucose, GlutaMAX™, pyruvate (Thermo Fisher Scientific, MA, United States of America) with 50% L-WRN-supernatant (ATCC^®^ CRL3276™ in DMEM, high glucose, GlutaMAX™, pyruvate plus 10% fetal calf serum), 1× N2 (Invitrogen, CA, United States of America), 1× B27 (Invitrogen, CA, United States), 50 ng/μL recombinant mouse epidermal growth factor (Sigma-Aldrich, MO, United States), 10 μM Y-27632 (Tocris, Bristol, United Kingdom), 1× Cellshield (Biochrom, Berlin, Germany)) was added and the cells were kept at 37°C, 5% CO_2_ and a humidified atmosphere. During the first few passages (2–3 weeks), remaining non-epithelial cells that were initially seeded together with epithelial organoid-forming cells gradually underwent cell death, as previously described by Chiu et al. for a similar culture model (Chiu et al., 2022). While organoid culture was accompanied by an adherent stromal cell monolayer, solely airway epithelial organoids grew within Cultrex embedding. These airway organoids were characterized by a basal cell layer positive for Trp63 and cytokeratin 5 (CK5), and a luminal CK8-positive differentiated cell layer including ciliated cells (acetylated alpha-tubulin-positive) (Supplementary Figure S1, Supplementary Video S1, Supplementary Files S1–S3).

### 2.2 Airway organoid culture and transduction

Airway organoids were cultured in growth medium at 37°C, 5% CO_2,_ and a humidified atmosphere as described above. Medium was changed every 2–3 days and organoids were passaged every 10–14 days. Genetic modification of organoid cells, allowing the expression of a fluorescent protein, was achieved via lentiviral transduction. Lentiviral particles were produced via third-generation split packaging protocol in HEK293T cells (DSMZ, Germany) as previously described (Schambach et al., 2006). The transfer plasmid contained the sequence for expression of a histone 2A-mCherry fusion protein under control of a Trp63 promoter, which was amplified from human genomic DNA as previously described (Lanza et al., 2006). Lentiviral transduction was performed based on a protocol previously described for employment in intestinal organoids by van Lidth de Jeude and colleagues (Van Lidth De Jeude et al., 2015).

### 2.3 Laser setup, imaging, and manipulation

For laser-based nanosurgery applications, including multiphoton imaging as well as manipulation, a Chameleon Ultra II laser system with a pulse length of 140 fs and a repetition rate of 80 MHz, previously described (Müller et al., 2019), was employed. Organoids labeled by histone 2A (H2A)-mCherry fusion protein were visualized at an excitation wavelength of 730 nm and emission was detected by a photomultiplier tube (Hamamatsu Photonics, Japan) using an emission filter at 607 ± 18 nm. Cell ablation was conducted with a wavelength of 730 nm, a pulse energy of 0.9 nJ, and a dissection speed of 15 μm/s.

### 2.4 Analysis of organoid morphology, cell proliferation, and migration

#### 2.4.1 Analysis of organoid morphology and cell migration

For analysis of organoid morphology and cell migration, organoids were transferred to a glass-bottom dish (µ-Dish 35 mm, high Grid-500 Glass, Ibidi, Germany). Therefore, Cultrex was dissolved and airway organoids were selectively pulled out of the suspension under a transmitted light microscope using a 20 µL pipette, followed by embedding into fresh Cultrex and covering with growth medium. The following day, the medium was changed to FluoroBrite™ DMEM (Thermo Fisher Scientific, MA, United States), and laser nanosurgery was conducted yielding ablation of either none (control), a single, two, five, or ten cells per organoid. Following cell ablation, two-photon microscopy images (z-stacks in the range of ±10 µm from the ablation plane with a step-size of 2 µm) were captured every 30 min over 4–6 h, and again at 24 h post-ablation. Organoids were kept under culture conditions (37°C, 5% CO_2_) throughout the experimental procedure.

For analysis of the impact of cell ablation on organoid morphology, organoid growth was quantitively determined using a custom Fiji macro calculating the mean organoid diameter. For this, the organoid’s minor and major axes on each of ten images captured per time point (ten z-slices in distances of 2 μm) were measured and subsequently averaged for each time point to obtain the diameter.

As airway organoids are characterized by a nearly centrosymmetric structure, we decided to quantify data using a 2D representation. Thus, for analysis of cell migration within organoids, maximum intensity projections of the captured z-stacks (±6 µm from ablation plane with a step-size of 2 µm) were generated for every point in time via Fiji. Using Fiji TrackMate plugin in combination with Stardist detector (Schmidt et al., 2018; Ershov et al., 2022), all cells were detected via their fluorescently labeled nuclei and subsequent employment of LAP Tracker identified the individual cells’ trajectories. To obtain data on every single cell’s migratory behavior in dependence on their distance from the ablation site, track data was further processed by a custom Matlab script.

#### 2.4.2 Analysis of cell proliferation

For analysis of cell proliferation within airway organoids, an EdU assay was performed as described previously by Donath et al. (2022). Briefly, organoids were transferred to a glass-bottom dish (µ-Dish 35 mm, high Grid-500 Glass, Ibidi, Germany) as described in Section 2.4.1. The following day, medium was changed to FluoroBrite™ DMEM (Thermo Fisher Scientific, MA, United States) supplemented with 10 µM 5-Ethynyl-2-deoxyuridine (5-EdU, Jena Bioscience, Germany) and organoids were incubated at 37°C, 5% CO_2_ for 2 h. Subsequent laser nanosurgery was conducted for ablation of either none (control), five or ten cells per organoid. At 4 h post cell ablation, organoids were fixed by incubation with 4% Roti®-Histofix (Carl Roth, Germany) supplemented with 1% glutaraldehyde (Carl Roth, Germany) for 20 min at RT. Cell permeabilization was conducted by incubation in 0.5% Triton X-100 (Carl Roth, Germany) in DPBS (Sigma-Aldrich, MO, United States) for 20 min at RT. A subsequent click-chemistry-mediated staining reaction was achieved by incubation of organoids in the reaction mix (1 mM Cu_2_SO_4_ (Jena Bioscience, Germany), 10 mM sodium ascorbate (Jena Bioscience, Germany), 8 µM 5-FAM Azide (Biomol, Germany) diluted in DPBS (Sigma-Aldrich, MO, United States)) for 1 h at 37°C in the dark, leading to fluorescent visualization of EdU that was incorporated into the DNA. Nuclear counterstaining was conducted using NucSpot^®^ 650 (final 1×, Biotium Inc., United States). Multi-channel z-stacks in the range of ±8 µm from the ablation plane with a step-size of 2 µm were captured by confocal laser scanning microscopy (Leica TCS SP5). As described in Section 2.4.1, for analysis, 2D representations of image data was used. Therefore, image data were processed using Fiji to generate a z-projection (sum slices), which was then used for nuclei detection of both EdU-positive as well as all cells via Fiji Stardist (Schmidt et al., 2018). The relative proliferation rate, defined as the ratio of EdU-positive to all cells, was determined and, using a custom Matlab script, was put into relation to the cells’ distances from the ablation site.

### 2.5 Transcriptome analysis via bulk RNA sequencing

For transcriptome analysis, 45 organoids were transferred to a glass-bottom dish (µ-Dish 35 mm, high Grid-500 Glass, Ibidi, Germany). The following day, medium was replaced by FluoroBrite™ DMEM (Gibco, United States), and laser-based ablation of ten neighboring cells within each organoid was conducted. Another dish with 45 organoids prepared analogously and kept under the same experimental conditions remained untreated as control. The organoids were incubated at 37°C, 5% CO_2_ and a humidified atmosphere for 4.5 h after cell ablation, followed by organoid harvesting using Cultrex^®^ 3D Culture Cell Harvesting Kit (Trevigen, MD, United States) according to the manufacturer’s protocol. After final centrifugation for 5 min at 850 × g and 4°C, organoids were resuspended in 1× DNA/RNA Shield™ (Zymo Research, CA, United States) and stored at −80 °C until all samples were collected. RNA isolation was conducted using *Quick*-RNA™ Microprep Kit (R1050, Zymo Research, CA, United States) following the manufacturer’s instructions. Subsequent library generation, RNA sequencing run, raw data processing, and differential expression analysis were performed by the Genomic core facility of Hannover Medical School.

#### 2.5.1 Library generation, quality control, and quantification

150 ng of total RNA per sample were utilized as input for mRNA enrichment procedure with ‘NEBNext^®^ Poly(A) mRNA Magnetic Isolation Module’ (E7490L; New England Biolabs) followed by stranded cDNA library generation using “NEBNext^®^ Ultra II Directional RNA Library Prep Kit for Illumina” (E7760L; New England Biolabs). All steps were performed as recommended in the user manual E7760 (Version 1.0_02-2017; NEB) except that all reactions were downscaled to 2/3 of initial volumes.

cDNA libraries were barcoded by dual indexing approach, using “NEBNext Multiplex Oligos for Illumina–96 Unique Dual Index Primer Pairs” (6440S; New England Biolabs). All generated cDNA libraries were amplified with 10 cycles of final PCR.

One additional purification step was introduced at the end of the standard procedure, using 1.2x “Agencourt^®^ AMPure^®^ XP Beads” (#A63881; Beckman Coulter, Inc.). Fragment length distribution of individual libraries was monitored using “Bioanalyzer High Sensitivity DNA Assay” (5,067-4626; Agilent Technologies). Quantification of libraries was performed by use of the “Qubit^®^ dsDNA HS Assay Kit” (Q32854; ThermoFisher Scientific).

#### 2.5.2 Library denaturation and sequencing run

Equal molar amounts of individually barcoded libraries were pooled for a common sequencing run in which each analyzed library constituted 16.7% of overall flowcell/run capacity. The library pool was denatured with NaOH and was finally diluted to 1.8 p.m. according to the Denature and Dilute Libraries Guide (Document # 15048776 v02; Illumina). 1.3 mL of the denatured pool was loaded on an Illumina NextSeq 550 sequencer using a Mid Output Flowcell (130 M cluster) for 2 × 75 bp paired-end reads (20024904; Illumina). Sequencing was performed with the following settings: Sequence reads 1 and 2 with 76 bases each; Index reads 1 and 2 with 8 bases each.

#### 2.5.3 BCL to FASTQ conversion

BCL files were converted to FASTQ files using bcl2fastq Conversion Software version v2.20.0.422 (Illumina).

#### 2.5.4 Raw data processing and quality control

Raw data processing was conducted by use of nfcore/rnaseq (version 1.4.2) which is a bioinformatics best-practice analysis pipeline used for RNA sequencing data at the National Genomics Infrastructure at SciLifeLab Stockholm, Sweden. The pipeline uses Nextflow, a bioinformatics workflow tool. It pre-processes raw data from FastQ inputs, aligns the reads and performs extensive quality-control on the results. The genome reference and annotation data were taken from GENCODE.org (*Mus musculus*; GRCm38. p6).

#### 2.5.5 Normalization and differential expression analysis

Normalization and differential expression analysis was performed on the internal Galaxy (version 20.05) instance of the RCU Genomics, Hannover Medical School, Germany with DESeq2 (Galaxy Tool Version 2.11.40.6) with default settings except for “Output normalized counts table”, which was set to “Yes” and all additional filters were disabled (“Turn off outliers replacement”, “Turn off outliers filtering”, and “Turn off independent filtering” set “Yes”). The different conditions were selected as primary factor whereas the batch was used as secondary factor in DESeq2 analyses (two factor design).

#### 2.5.6 Gene ontology enrichment analysis

Gene ontology enrichment analysis was performed on the basis of all DEGs (p-adj <0.05) using Cytoscape software (version 3.9.1) (Shannon et al., 2003) and StringApp (Doncheva et al., 2019).

### 2.6 Image analysis and statistics

All image data analysis was performed via Fiji (Schindelin et al., 2012), including Stardist (Schmidt et al., 2018) and TrackMate (Ershov et al., 2022) plugins. Further data analysis, including graphical representation, was conducted using Matlab software (version 2022a) and OriginPro 2021b (version 9.8.5.201, OriginLab Corporation, Northampton, MA, United States).

Statistical analysis was performed using Microsoft Excel (Microsoft Corporation, Redmond, WA, United States). Student’s unpaired *t*-test and One-Way ANOVA were used to test for significant differences between groups, thereby applying a significance level of *α* = 0.05.

## 3 Results

### 3.1 Local cell death has no consequences for organoid survival and long-term morphology

As described by Donath et al., native colonic epithelial repair processes can be observed in colonoids on a cellular level upon femtosecond laser-based ablation of as little as a single cell (Donath et al., 2022). Based on this, we aimed to generate an airway organoid damage model allowing for the examination of endogenous repair mechanisms on a single-cell level, triggered by highly localized injury induction via ablation of few cells.

Initially, to study how local cell death affects the overall viability and morphology of airway organoids, femtosecond laser-mediated damage was induced on a single-cell level, and organoids were tracked using multiphoton microscopy over a subsequent period of 24 h. Untreated organoids kept under the same experimental conditions served as control.

**FIGURE 2:**
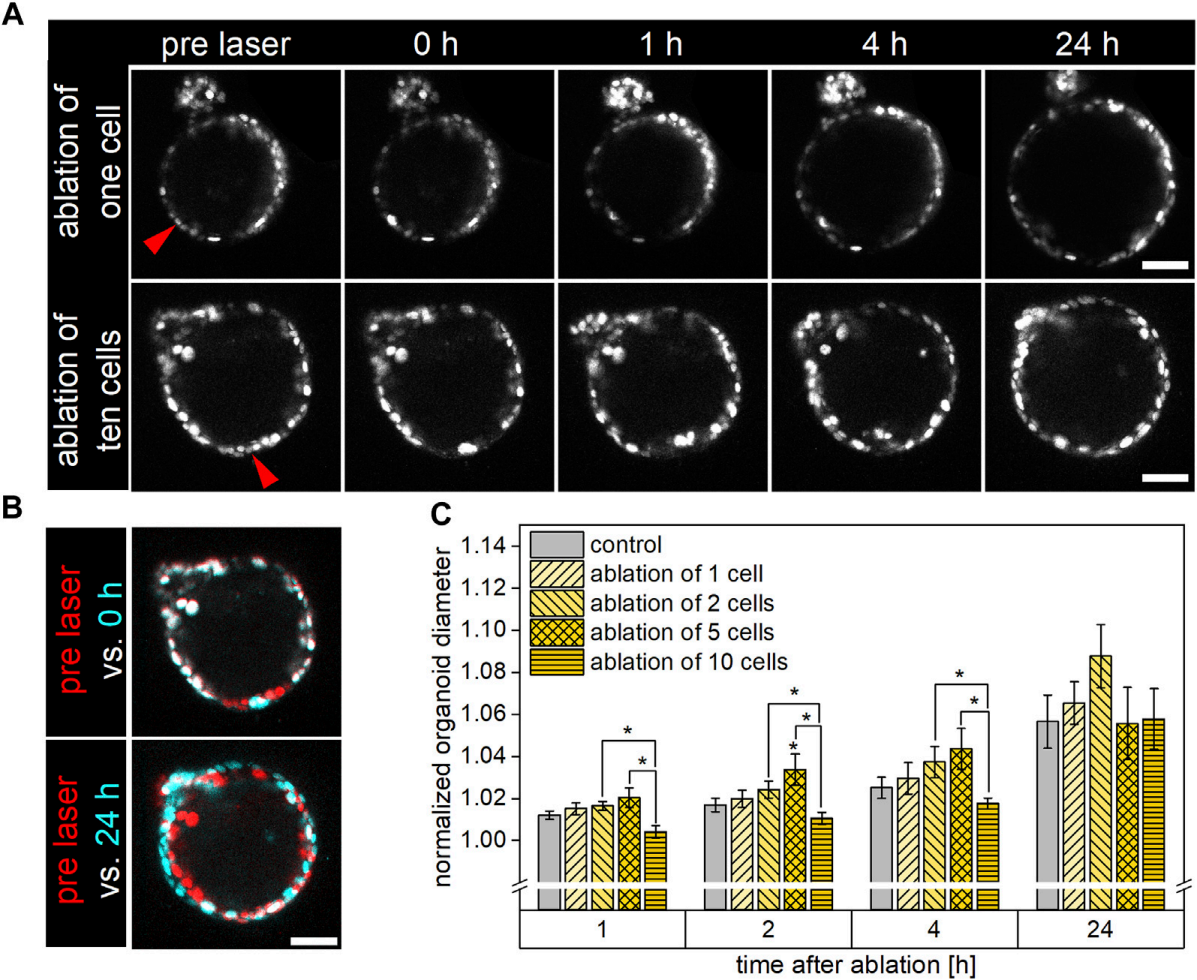
Influence of single or multiple cell ablation on morphology, structural integrity, and growth behavior of airway organoids. The organoids’ structural integrity was restored within 24 h post cell ablation independent of the extent of damage. Organoids subjected to the ablation of five cells showed an increased growth potential in the early phase after damage induction, while ablation of ten cells tended to decelerate organoid growth within this time. **(A)**: Representative timelines showing organoids pre, as well as 0, 1, 4, and 24 h post ablation of either a single (top row) or ten neighboring (bottom row) cells. Red arrows indicate ablation site. Scale bar: 50 µm. **(B)**: Color-labeled overlays of organoid images captured pre (red) and post (cyan) laser manipulation. Immediately following cell ablation (top), red- and cyan-colored cells indicate the damage area, in which the cyan-colored signal originates from strong autofluorescence of ablated cells. At 24 h post laser ablation (bottom), the organoid had grown, indicated by non-overlapping red and cyan color-labeled cells. Scale bar: 50 µm. **(C)**: Relative diameter of control organoids as well as of organoids subjected to ablation of either a single, two, five, or ten cells at 1, 2, 4, and 24 h post laser manipulation. Data shown represent mean ± SEM, *n* ≥ 6 per condition, *: *p* < 0.05, statistics refer to control if not stated otherwise.

Upon ablation of either a single or multiple (two, five, or ten) neighboring cells, the overall organoid viability within 24 h post cell ablation, determined by microscopic examination of the organoid’s structural integrity, was closely 100% and did not differ from the survival rate observed in control organoids (Figure 2). For either condition, cell ablation immediately led to a strong autofluorescence signal within the damage site. Subsequently, a shedding of the dead cell/s into the organoid’s lumen and an invagination of cells surrounding the damaged area was observed within 1 h. While the injured area showed at least a partial repair at 4 h post laser-based ablation, especially in the case of ablation of five or ten cells, the damage site was still clearly visible at this point in time (Figure 2A). Independent of the extent of damage, the observed gap within the cell layer was closed within 24 h after laser treatment, the structural integrity was restored. Representative timelines of organoids subjected to ablation of either a single or ten cells, captured in the period from pre until 24 h post ablation via multiphoton microscopy, are shown in Figure 2A. Color-labeled image overlays of the organoid pre laser treatment (red) and either of the subsequent captures (cyan) served for better visualization of the damage site and potential morphological changes in the course of repair (Figure 2B).

For examination of the impact of cell ablation on organoid morphology, organoid growth was quantitively analyzed using a custom Fiji macro that determined the mean organoid diameter. For this, the organoid’s minor and major axes on each of the ten images captured per time point (ten z-slices in distances of 2 µm) were measured and subsequently averaged for each time point to obtain the diameter. For these experiments, organoids with an initial diameter of 148 ± 4 µm were used, and growth to a mean diameter of 157 ± 5 µm was observed within 24 h. For further analysis and better comparison between the groups, all raw data were normalized to the respective organoid’s initial size pre ablation.

The changes over time of the normalized organoid’s mean diameters of each group are depicted in Figure 2C. It was found that, within 24 h, the organoids’ diameters of the different conditions increased by 5.7% ± 1.3%, 6.5% ± 1.0%, 8.8% ± 1.5%, 5.6% ± 1.7% and 5.8% ± 1.4% in case of control, ablation of a single, two, five, and ten cells, respectively, with no statistically significant differences between any two conditions. In the early period of repair, especially ablation of five cells slightly promoted the organoids’ growth potential compared to the control. In this context, control organoid diameter increases to 101.2% ± 0.2%, subsequently to 101.7% ± 0.3%, and further to 102.5% ± 0.5% after one, two, and 4 h were measured. In comparison, organoids subjected to ablation of five cells had grown to 102.0% ± 0.5% (*p* = 0.09), 103.4% ± 0.7% (*p* = 0.03), and 104.4% ± 1.0% (*p* = 0.19) of their original diameter at the same time points. On the contrary, ablation of ten cells seemed to arrest organoid growth within the first 4 h post laser treatment. In this case, only minor increases in organoid diameter to 100.4% ± 0.3% (*p* = 0.08) and subsequently to 101.1% ± 0.3% and to 101.8% ± 0.3% after one, two, and 4 h after ablation were determined. These changes in size were less than those observed for the control group (*p* > 0.05) as well as for organoids subjected to two- or five-cell-ablation (*p* < 0.05).

In conclusion, laser-based ablation of up to ten cells did not affect the overall viability or mid-term (24 h) growth potential of airway organoids, while enhanced or decelerated organoid size increases were observed in the early repair period in case of ablation of five or ten cells, respectively.

### 3.2 Cell death leads to increased proliferation in airway organoids dependent on the extent of damage

Next, we aimed for a more detailed investigation of cell proliferation—a major contributing factor of organoid growth and epithelial restitution (Crosby and Waters, 2010)—within organoids following laser-based nanosurgery. For this, an EdU assay was performed to fluorescently label cells that underwent cell cycle S-phase within 4 h post cell ablation (Kotogány et al., 2010). As previous morphological analyses revealed that ablation of five or ten cells led to the most pronounced alterations in organoid growth behavior within 4 h post laser treatment, these conditions were chosen to examine the proliferation of individual cells within airway organoids. Organoids kept under the same experimental conditions without being subjected to laser nanosurgery served as control.

**FIGURE 3:**
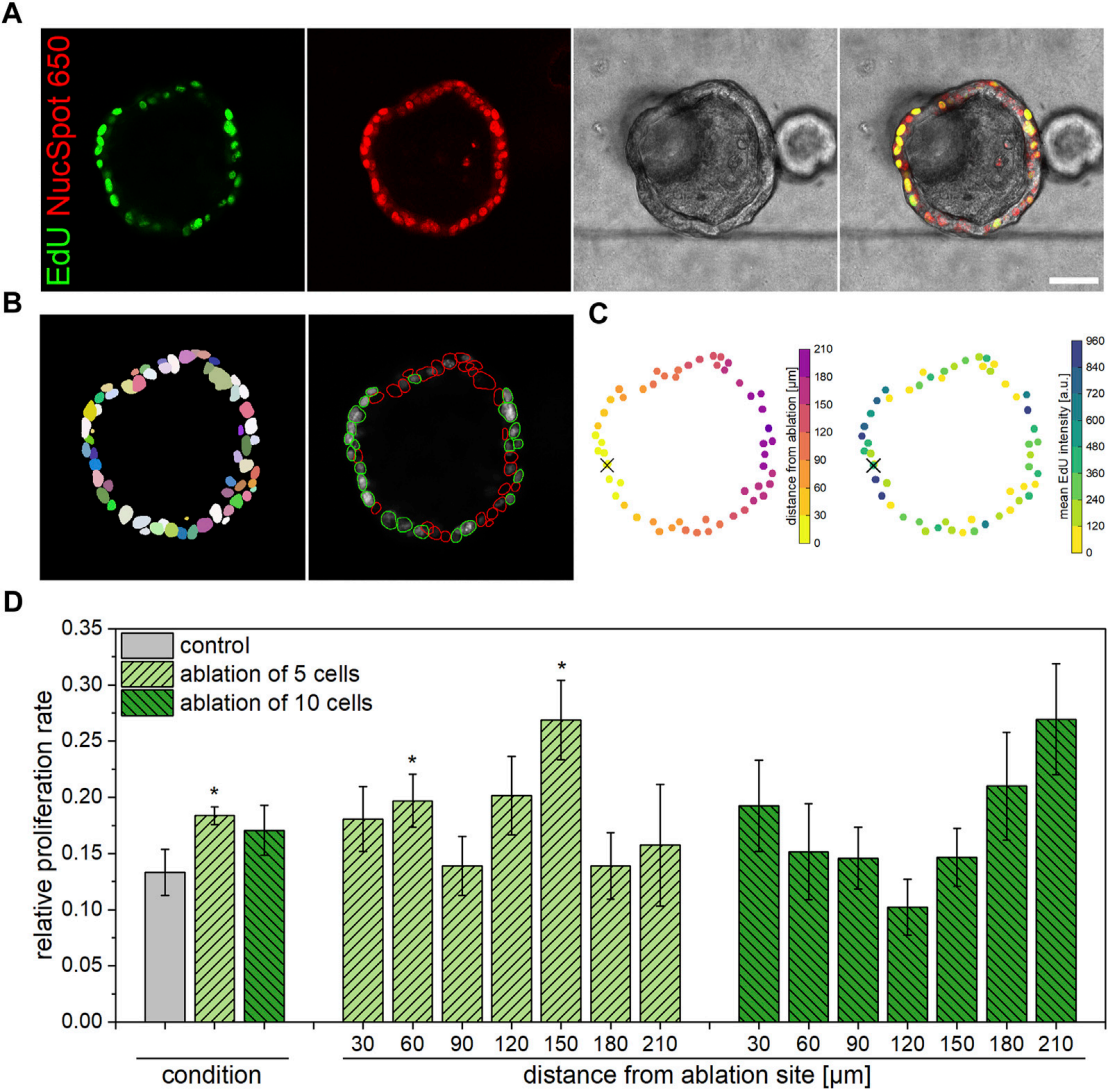
Influence of multiple cell ablation on proliferation within airway organoids. Ablation of five cells induced an enhanced proliferative behavior within 4 h post laser nanosurgery in cells within close proximity as well as at an intermediate distance to the ablation site. **(A)**: Representative confocal microscopy images showing an organoid 4 h post ablation of ten cells, stained for EdU incorporation (green) and all nuclei (red). Scale bar: 50 µm. **(B)**: Images obtained from Fiji analysis workflow showing detection of all cells via Stardist (left) and subsequent determination of EdU-positive (green circling) vs. all cells (red circling) (right). **(C)**: Images obtained from Matlab analysis workflow showing the distance from ablation site (left) and the mean EdU staining intensity (right) of each cell. Cross indicates center of ablation site. **(D)**: Relative location-independent (left) and location-dependent (right) proliferation rate of control organoids as well as of organoids subjected to ablation of either five or ten cells at 4 h post laser manipulation. Data shown represents mean ± SEM, *n* ≥ 8 per condition, *: *p* < 0.05, statistic refers to control if not stated otherwise.

Qualitatively, confocal microscopy images of fixed organoids stained for EdU incorporation with nuclear counterstain show the extent of proliferative cells within each organoid, representatively illustrated in Figure 3A. For quantitative analysis of the relative proliferation rate within each organoid, Fiji Stardist plugin (Schmidt et al., 2018) was employed to recognize all as well as EdU positive cells only (Figure 3B) in images acquired via confocal microscopy. A subsequent application of a custom MatLab script fitted all organoid cells to a circle, in which the distance from the ablation site of each cell was determined (Figure 3C). Further, the script supplied data on the relative proliferation rate in dependence on the distance from the ablation site for each condition. As presented in Figure 3D, it was found that the overall proliferation rate in organoids subjected to ablation of five cells amounts to 18.4% ± 0.8% at 4 h post laser treatment, which represents a significant increase in comparison to untreated organoids (13.3% ± 2.1%, *p* = 0.04). It can further be observed that, in the case of ablation of five cells, an enhanced number of proliferative cells is located either in close proximity to or in an intermediate distance to the ablation site. In this context, proliferation rates of 19.7% ± 2.4% and 26.9% ± 3.5% were measured at distances from the ablation site of 30–60 μm and 120–150 μm, respectively. However, compared to the control, no significant alterations of the proliferation rate were detected for organoids subjected to ten-cell-ablation in either case of overall or location-dependent analysis. Against expectations gained from previous morphological analyses, the location-independent proliferation rate of organoids in which ten cells were ablated amounted to 17.1% ± 2.2%, which was slightly higher than the one determined for control organoids (*p* = 0.18). Also, similar to the observations made for organoids subjected to ablation of five cells, tendencies for enhanced proliferation rates in direct proximity to the ablation site (19.2% ± 4.1% at distances of 0–30 μm, *p* = 0.13) as well as further away (26.9% ± 4.9% at distances of 180–210 μm, *p* = 0.05) were detected in organoids after ablation of ten cells.

In conclusion, it was found that especially ablation of five cells induced an enhanced proliferation rate within airway organoids in the early phase of damage repair. In this context, the observed broad induction of cell proliferation is attributable to locally significantly increased proliferation events in cells in close proximity to as well as in an intermediate distance from the ablation site.

### 3.3 Large-area damage results in increased localized migration

**FIGURE 4:**
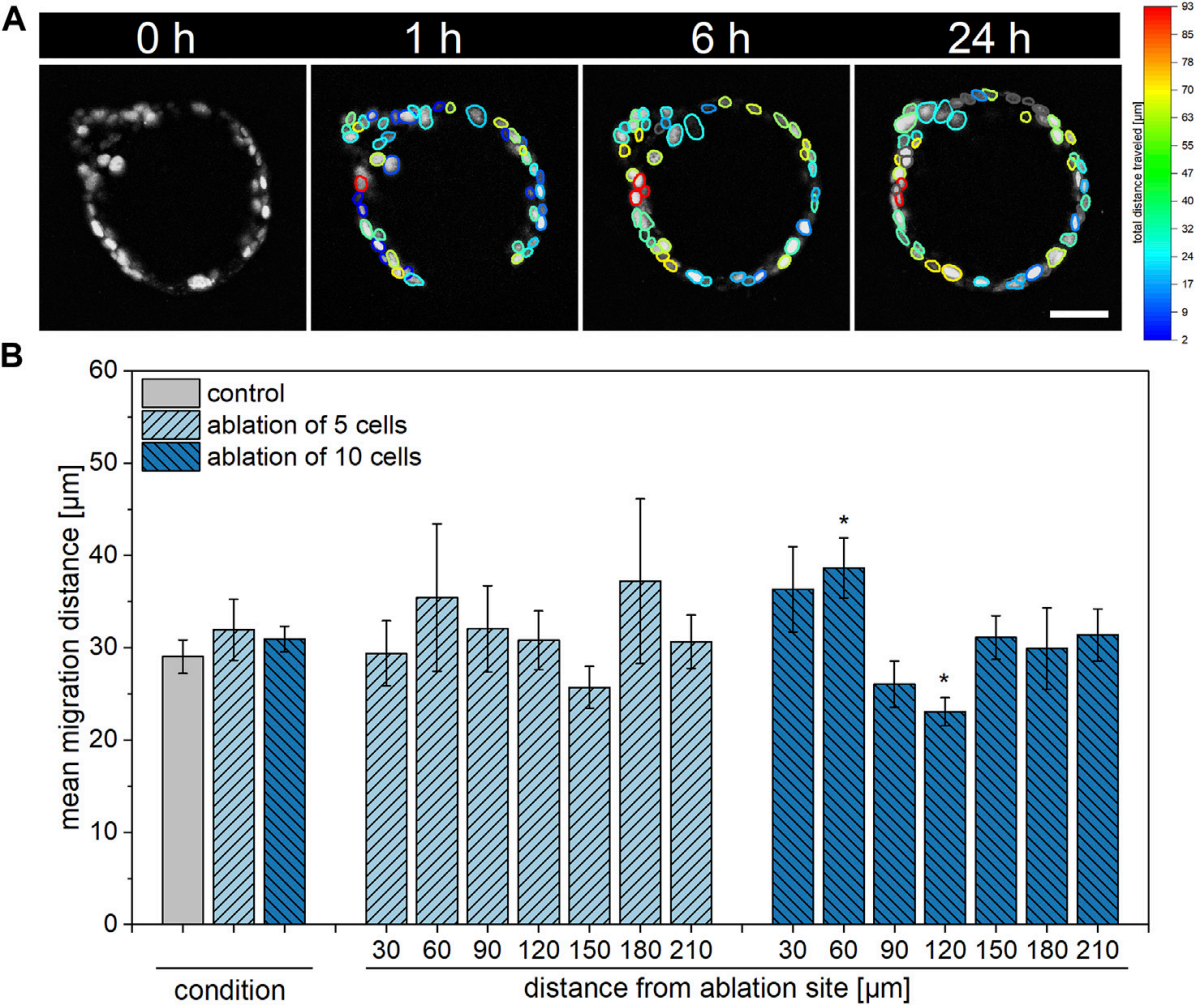
Influence of multiple cell ablation on single cell migration distance within airway organoids. Alterations in migratory behavior of cells in close proximity to or at an intermediate distance from the ablation site were detected in organoids subjected to ablation of ten cells. **(A)**: Representative timeline showing an organoid 0, 1, 4, and 24 h post ablation of ten cells with visualization of each cell’s total distance travelled as detected by TrackMate. Scale bar: 50 µm. **(B)**: Relative location-independent (left) and location-dependent (right) mean migration distance of control organoids as well as of organoids subjected to ablation of either five or ten cells at 24 h post laser manipulation. Data shown represents mean ± SEM, *n* ≥ 8 per condition, *: *p* < 0.05, statistic refers to control if not stated otherwise.

As epithelial restitution generally depends on both cell proliferation as well as migration (Zahm et al., 1997), we next aimed to examine the migratory behavior of organoid cells within 24 h post laser treatment. For this, images of organoids subjected to ablation of either no (control), five or ten cells acquired via multiphoton microscopy were analyzed using Fiji Stardist (Schmidt et al., 2018) and Trackmate (Ershov et al., 2022) plugins. The achieved track results, illustrated as the maximum distance traveled in Figure 4A, were employed in a custom Matlab script. Analogous to the handling of EdU data, the script fitted all organoid cells to a circle, in which the distance from the ablation site of each cell was determined and data of the migrated distance of each cell in dependence on the distance from the ablation site was supplied.

As represented in Figure 4B, organoid cells of all conditions showed similar migration behavior when analyzed location-independently with mean migration distances of 29.1 ± 1.8 µm, 32.0 ± 3.3 µm and 30.9 ± 1.4 µm within control organoids or after ablation of five or ten cells, respectively, within 24 h. Upon ablation of five cells, migratory activity was not found to be locally enhanced or reduced at 24 h post laser treatment in a significant manner. On the contrary, in comparison to the control, ablation of ten cells led to an increased migration distance of 38.7 ± 3.2 µm of cells within a distance of 30–60 µm from the ablation site (*p* = 0.02). Further, a decrease in migration distance to 23.1 ± 1.5 µm was observed in cells within an intermediate distance from the ablation site of 90–120 µm (*p* = 0.02).

Taken together, alterations in migratory behavior of cells were found in a location-dependent manner in response to ablation of ten, but not five cells.

### 3.4 Transcriptome analysis reveals the influence of cell ablation on migration, differentiation, and further developmental processes next to cell death and stress pathways

**FIGURE 5:**
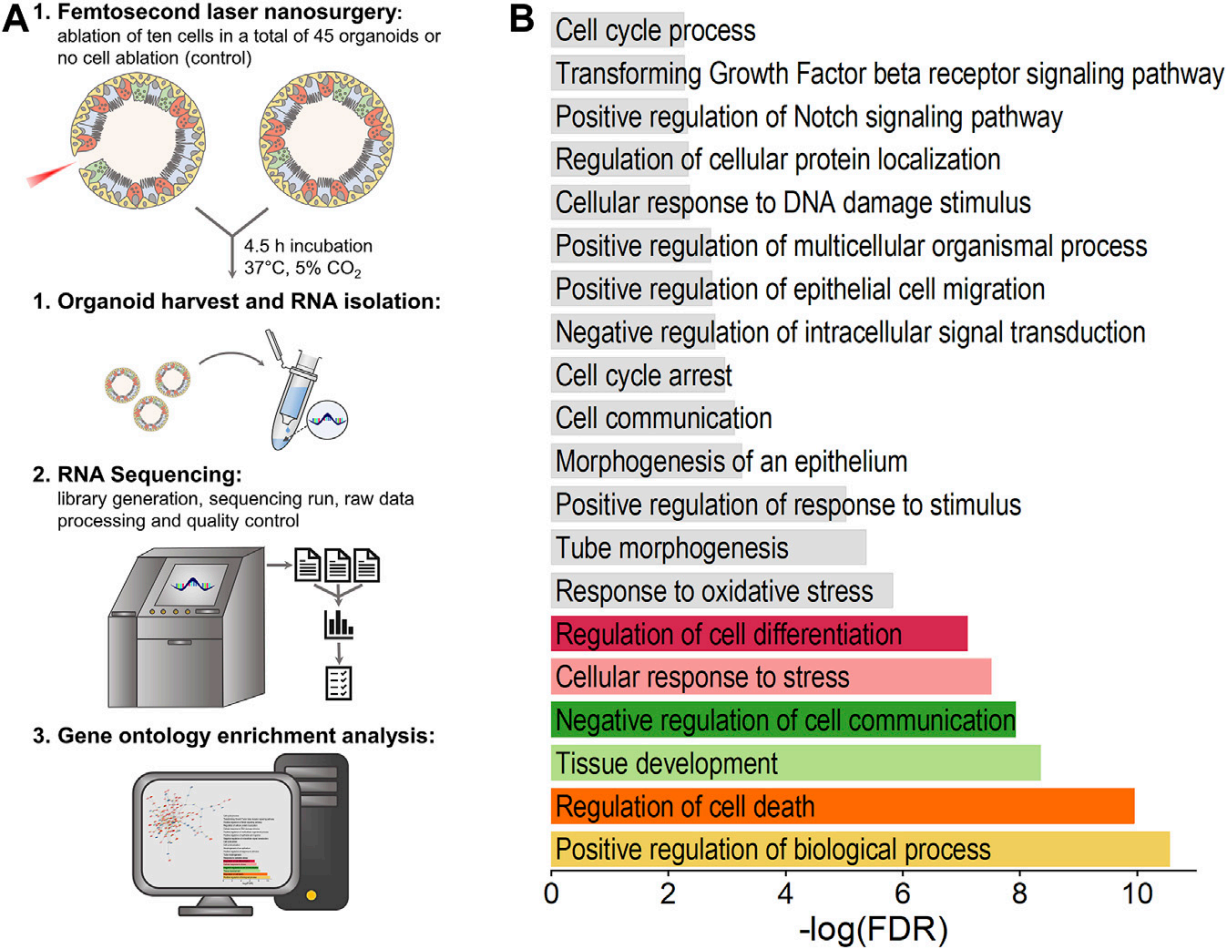
Influence of ablation of ten cells on airway organoids’ transcriptome. Both genes related to damage induction as well as to repair mechanisms were found to be differentially expressed. **(A)**: Schematic illustration of experimental procedure for transcriptome analysis. **(B)**: Biological processes significantly affected by laser treatment as determined by gene ontology enrichment analysis using STRING database. Data analysis was performed on the basis of RNA-seq data obtained from *n* = 6 samples per group.

To gain further insights into the functional processes affected by laser-based damage induction and subsequent repair mechanisms, we compared the transcriptomes of organoid cells of untreated condition and post cell ablation via bulk RNA sequencing. According to previous results, which indicated both migration as well as proliferation as driving processes of repair in the case of ablation of ten cells, this condition was chosen for RNA sequencing. A schematic representation of the experimental procedure is shown in Figure 5A.

In relation to the total number of cells an organoid is composed of, ablation of ten cells represents a rather small and highly localized injury. Thus, we expected that, primarily, cells in close proximity to the damage site are involved in early repair processes, which would lead to relatively small changes in the overall measured expression profiles of treated *versus* untreated organoids. We therefore decided to include all differentially expressed genes (DEGs) with a p-adjusted <0.05 for analysis, independently of their determined fold change. In this context, we identified a total of 218 mRNA transcripts, including 129 upregulated and 89 downregulated transcripts, that were differentially expressed in the laser-treated group compared to the control. Notably, these DEGs included *Trp63* (p-adj = 4.79E-9) and *Sox2* (p-adj = 2.54E-2), both of which play a pivotal role in the maintenance of airway epithelial basal cells (Ochieng et al., 2014), while *Krt8* as the gene coding for a general marker of luminal cell types of the airway epithelium (Rock et al., 2009) was not found to be significantly dysregulated (FC = 0.10, p-adj = 0.26).

Subsequent gene ontology (GO) enrichment analysis using STRING database was performed based on all DEGs, which yielded 85 significantly enriched GO terms for biological processes (FDR <0.05), partially displayed in Figure 5B. It was found that, on the one hand, the analyzed DEGs are involved in the positive regulation of biological process (FDR = 2.80E-11, 73 genes, e.g., *Cdc45*, *Tmprss2*, *Plin2*, *Pabpc1*, *Ier3*), regulation of cell death (FDR = 1.13E-10, 37 genes, e.g., *Ier3*, *Nqo1*, *Robo1*, *Xdh*, *Cyp1b1*), cellular response to stress (FDR = 3.12E-8, 31 genes, e.g., *Cdc45*, *Ier3*, *Nqo1*, *Atf6b*, *Robo1*) and response to oxidative stress (FDR = 1.49E-6, 15 genes, e.g., *Nqo1*, *Cyp1b1*, *Pycr1*, *Pcna*, *Slc7a11*), which can all be directly linked to damage induction. On the other hand, the GO biological processes of tissue development (FDR = 4.53E-9, 35 genes, e.g., *Lgr5*, *Vcl*, *Robo1*, *Cyp1b1*, *Hbegf*), regulation of cell differentiation (FDR = 7.96E-8, 34 genes, e.g., *Ptprg*, *Vcl*, *Robo1*, *Xdh*, *Ddit3*), tube morphogenesis (FDR = 4.19E-6, 19 genes, e.g., *Lgr5*, *Robo1*, *Cyp1b1*, *Hbegf*, *Epha7*) and positive regulation of epithelial cell migration (FDR = 1.80E-3, 7 genes, e.g., *Hbegf*, *Ptgs2*, *Tgfb2*, *Sema5a*, *Plcg2*) were determined to be significantly enriched upon laser-based cell ablation. Further, enrichment analysis identified overall cell communication (FDR = 7.50E-4, 43 genes, e.g., *Ier3*, *Stk38*, *Atf6b*, *Lgr5*, *Robo1*) as well as Notch (FDR = 4.70E-3, 4 genes, *Robo1*, *Tgfb2*, *Sox2*, *Trp63*) and transforming growth factor beta (TGF-beta) receptor (FDR = 5.30E-3, 5 genes, *Ltbp4*, *Tgfb2*, *Jun*, *Trp53*, *Skil*) signaling pathways as specifically regulated in response to laser treatment. The involvement of genes in the top six significant GO biological processes as well as their fold-change determined by RNA-seq is illustrated in Supplementary Figure S2.

In conclusion, despite the presumably minor number of cells involved in the early repair processes of airway organoids upon laser-based ablation, changes in expression of genes related to both damage induction as well as cellular regenerative responses were detected.

## 4 Discussion

Especially chronic pulmonary diseases can cause severe damage in airway epithelial tissue, often finally leading to conditions, in which endogenous regeneration mechanisms fail to repair (Rock and Königshoff, 2012; Barkauskas et al., 2017). Unfortunately, effective medication targeting early cellular injury responses, thereby supporting the cells’ native homeostasis and repair programs, have yet to be developed as the underlying mechanisms are still not fully understood (Barkauskas et al., 2017). To enable studying such mechanisms on the long-term, we established an *in vitro* damage model which enables the precise application of injury to airway epithelial cells within self-assembled organoids, allowing for subsequent tracking of triggered repair processes in a close-to-native, multicellular 3D environment via state-of-the-art microscopy techniques.

For the characterization of early repair processes induced in airway organoids upon targeted ablation of a single or up to ten cells, the effects of femtosecond laser-based manipulation on the organoids’ structural integrity as well as the cells’ migratory and proliferative behavior were studied. Airway epithelial tissue is described to unleash a remarkable endogenous regeneration potential upon injury (Rao Tata and Rajagopal, 2017). Based on this, we expected a fast and efficient repair of the gap within the cell layer restoring the epithelium’s structural integrity, induced by targeted ablation. This was observed in nearly all studied organoids (within 4–10 h post laser-based injury infliction). The obtained data show that both migration and proliferation are potentially involved in the airway organoids’ repair process following laser-based nanosurgery. This indicates that targeted cell ablation might induce a cellular program with similar mechanisms occurring during epithelial restitution upon injury in native lung tissue (Crosby and Waters, 2010). It was shown that the employed airway organoids are generally characterized by a consistent growth behavior, which is in good agreement with published data of similar cultures (Rabata et al., 2017; Sachs et al., 2019; Ekanger et al., 2022). Still, the underlying enhanced proliferation rate compared to the native tissue’s properties needs to be taken into consideration when employing this model for regeneration studies. In this context, on the one hand, the data suggest that ablation of five cells or less, posing a very small damage area, results in a fast repair dominated by early cell proliferation. On the other hand, cell cycle progress might be paused in an early phase upon ablation of ten cells, thereby enabling migration of nearby cells to take part in restoring the epithelial barrier. Notably, according to previous publications, one of the first and also most important processes that characterize repair of injured airway epithelial tissue is cell migration rather than proliferation (Erjefält et al., 1995; Zahm et al., 1997; Puchelle et al., 2006). Thus, the sequence of events occurring upon ablation of ten cells mimics the described procedure of epithelial wound healing, marking this as a potentially suitable model for studying early repair processes *in vitro*.

The results obtained by image analysis are further supported by the transcriptomic changes within airway organoids upon laser-based ablation of ten cells revealed by RNA-seq, which indicate the enrichment of the processes of cell cycle arrest and positive regulation of epithelial cell migration in the early phase of repair. Notably, also a variety of genes directly linked to epithelial repair were found to be differentially expressed. Among these, we found *Trp63* to be significantly upregulated upon cell ablation. It was previously shown that *Trp63* acts as a positive regulator of a set of genes functionally associated with epithelial differentiation and repair and necessary for wound healing (Warner et al., 2013). In good agreement with Warner et al., the described target genes such as *CTNNB1* (beta-catenin), *EGFR,* and *JAG1* (Jagged1) were not determined to be significantly differentially expressed, potentially attributable to a saturation of Trp63 signaling (Warner et al., 2013). Also, an increase in the number of basal cells, of which the expression of *Trp63* is a major characteristic, could have contributed to the determined upregulation. Since, in accordance with the EdU assay, we did not find cell proliferation to be specifically enhanced after the ablation of ten cells, dedifferentiation processes of secretory cells might be involved in the restoration of the epithelial barrier. In this context, YAP-1 was described to act as an important transcriptional coactivator regulating the occurrence of a dedifferentiation program in secretory cells and would be an interesting candidate to focus on in further studies (Zhao et al., 2014). As the mechanism of secretory cell dedifferentiation has only been observed after the elimination of a majority of the basal cell population though (Rawlins et al., 2009; Tata et al., 2013), further experiments will be necessary to evaluate whether localized cell ablation analogously induces local dedifferentiation effects.

The positive regulation of Notch signaling pathway, which was determined to be enriched upon cell ablation, might have different effects. On the one hand, Notch signaling was reported to support the maintenance of basal cells (Carraro et al., 2020), while on the other hand, Pardo-Saganta et al. found that basal cells support the maintenance of their progenies’ secretory cell state also by Notch signaling (Pardo-Saganta et al., 2015).

Moreover, we showed that TGF-beta signaling was enriched in airway organoids upon targeted damage induction. TGF-beta is generally known to contribute to various regulatory functions including cell growth, migration, and differentiation (Zhao and Young, 1996; Beck et al., 2003). The physiological production of TGF-beta in airway epithelial cells under homeostatic conditions was shown to increase upon injury, suggesting that cytokine plays a role in wound healing (Thompson et al., 2006). While Thompson et al. demonstrated that TGF-beta2 contributes to the modulation of subepithelial extracellular matrix homeostasis (Thompson et al., 2006), Ito et al. further described TGF-beta as a stimulator of airway epithelial repair after mechanical injury (Ito et al., 2011). In this context, it was found that TGF-beta induces the production of HB-EGF in airway epithelial cells, which subsequently leads to phosphorylation of EGFR and thus autocrine activation of EGF signaling as key factor in the repair process (Ito et al., 2011). In good agreement with this, the expression of both TGF-beta2 and HB-EGF were determined to be upregulated in airway organoids upon cell ablation.

In conclusion, the employment of femtosecond laser-based nanosurgery for targeted cell ablation in airway organoids poses a novel approach for the investigation of cellular damage responses in the course of early epithelial repair after cellular lung injury. This method creates the possibility to reduce animal experiments and have a fine tempo-spatial resolution of the repair process and underlying mechanisms of airway epithelium. We showed that ablation of ten neighboring cells induces a repair program including cell migration and subsequent proliferation in airway organoids, thereby potentially resembling the mechanisms of epithelial restitution in native tissue. Further functional analyses revealed the involvement of Notch and TGF-beta signaling activity previously described to regulate epithelial repair in airways. Based on these findings, future experiments utilizing a lentiviral Notch signaling activity reporter allowing for the tracking of dynamic signaling levels in individual cells via live imaging are of high interest.

Together, the herein described *in vitro* 3D damage model of airway organoids can be employed to further enlighten the intra- and intercellular mechanisms induced by local injury and necessary for efficient epithelial repair. By combining the culture of airway epithelial cells with, e.g., immune cells such as macrophages, an even more complex model system can be designed and applied for translational studies.

## Data Availability

The datasets presented in this study can be found in online repositories. The names of the repository/repositories and accession number(s) can be found below: NCBI GEO, under GSE241237.
