# Supplementary figures and images for: Mimicking acute airway tissue damage using femtosecond laser nanosurgery in airway organoids
Source: Front Cell Dev Biol. 2023 Sep 8;11:1268621. doi: 10.3389/fcell.2023.1268621 (PMC10514509; doi:10.3389/fcell.2023.1268621)

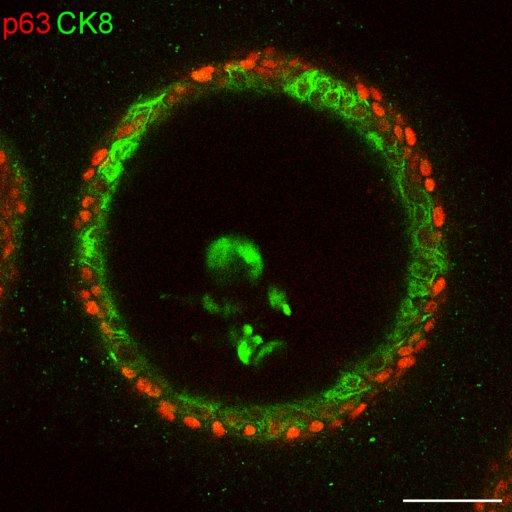

Supplement: Supplementary file 2 [file Presentation2.zip › Supplementary File 2.TIF]

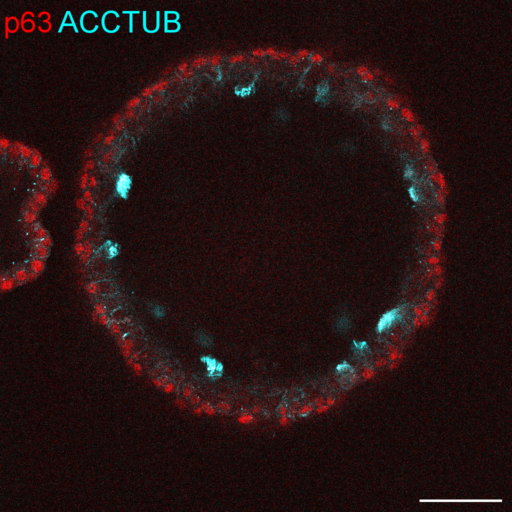

Supplement: Supplementary file 3 [file Presentation3.zip › Supplementary File 3.TIF]
