# Supplementary material for: Mimicking acute airway tissue damage using femtosecond laser nanosurgery in airway organoids
Source: Front Cell Dev Biol. 2023 Sep 8;11:1268621. doi: 10.3389/fcell.2023.1268621 (PMC10514509; doi:10.3389/fcell.2023.1268621)
Supplement: Supplementary file 4 [file DataSheet1.PDF]

Supplementary Material

Mimicking acute airway tissue damage using femtosecond laser nanosurgery in airway organoids

Lara Gentemann[1,2,3,*], Sören Donath[1,2], Anna E. Seidler[1,2], Lara Patyk[1,2], Manuela Buettner[4], Alexander Heisterkamp[1,2,3,5], Stefan Kalies[1,2,3,5,*]

[1]Institute of Quantum Optics, Leibniz University Hannover, Hannover, Germany

[2]Lower Saxony Center for Biomedical Engineering, Implant Research and Development, Hannover, Germany

[3]REBIRTH Research Center for Translational Regenerative Medicine, Hannover, Germany

[4]Institute for Laboratory Animal Science, Hannover Medical School, Hannover, Germany

[5]German Center for Lung Research (DZL), Gießen, Germany

*** Correspondence:** gentemann@iqo.uni-hannover.de and kalies@iqo.uni-hannover.de

1 Supplementary Methods

1.1 Immunofluorescence of whole mount airway organoids

Airway organoids grown on a glass-bottom dish (µ-Dish 35 mm, high Grid-500 Glass, Ibidi, Germany) were washed with DPBS (Sigma Aldrich, MO, USA) three times followed by incubation with fixing solution (4 % paraformaldehyde in DPBS) for 30 minutes at RT. Subsequently, fixing solution was removed, organoids were washed with DPBS three times and incubated in permeabilization/quenching solution (125 mM glycine, 0.5 % Triton X-100 in DPBS) for 30 minutes at RT, followed by incubation in blocking solution (5 % FBS, 0.25 % Triton X-100, 0.5 % Tween-20 in DPBS) for 1 hour at RT. Subsequently, organoids were incubated with primary antibody diluted in blocking solution for 1 hour at RT and then overnight at 4 °C. The next day, organoids were washed with DPBS three times for 5-10 minutes each, followed by incubation with secondary antibody diluted in blocking solution for 2 hours at RT in the dark. Finally, organoids were washed with DPBS three times for 5-10 minutes each. Stained organoids were covered with DPBS and analyzed using a confocal laser scanning microscope (Leica TCS SP5). Primary antibodies: mouse-anti-acetylated-alpha-tubulin (1:200 6-11B-1, Santa Cruz Biotechnology), mouse-anti-CK5 (1:200 2C2, Invitrogen), rat-anti-CK8 (1:100 TROMA-I, DSHB), rabbit-anti-p63 (1:200 Poly6190, BioLegend); secondary antibodies: m-IgG-kappa-BP-CFL-488 (1:200, Santa Cruz Biotechnology), donkey-anti-rat-IgG-(H+L)-Alexa-Fluor-Plus-647 (1:200, Invitrogen), donkey-anti-rabbit-IgG-Alexa-Fluor-647 (1:200 Poly4064, BioLegend), goat-anti-mouse-IgG-Alexa-Fluor-488 (1:200, Invitrogen).

2 Supplementary Figures, Videos and Files

2.1 Supplementary Figures

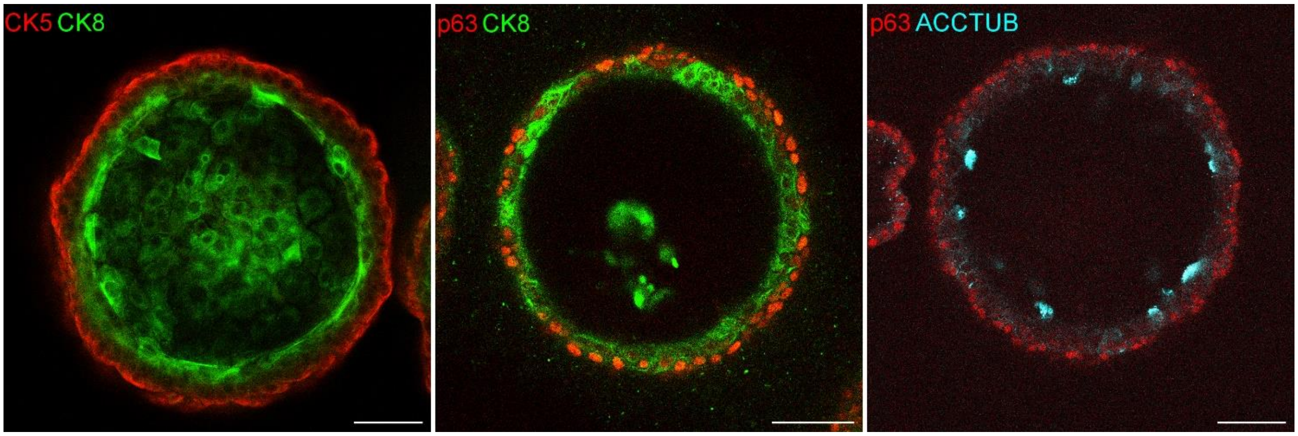

Supplementary Figure S1: Immunofluorescence staining of airway organoids. Airway organoids are composed of an outer layer of CK5-positive (red, left), p63-positive (red, center/right) basal cells and a luminal layer of CK8-positive (green, left/center) differentiated airway epithelial cells, some of which contain ACCTUB-positive (cyan, right) cilia. Please refer to supplementary Video S1 and supplementary Files S1-S3 for underlying 3D data. ACCTUB: acetylated alpha-tubulin; CK5: cytokeratin 5; CK8: cytokeratin 8; p63: transformation related protein 63. Scale bars: 50 µm.

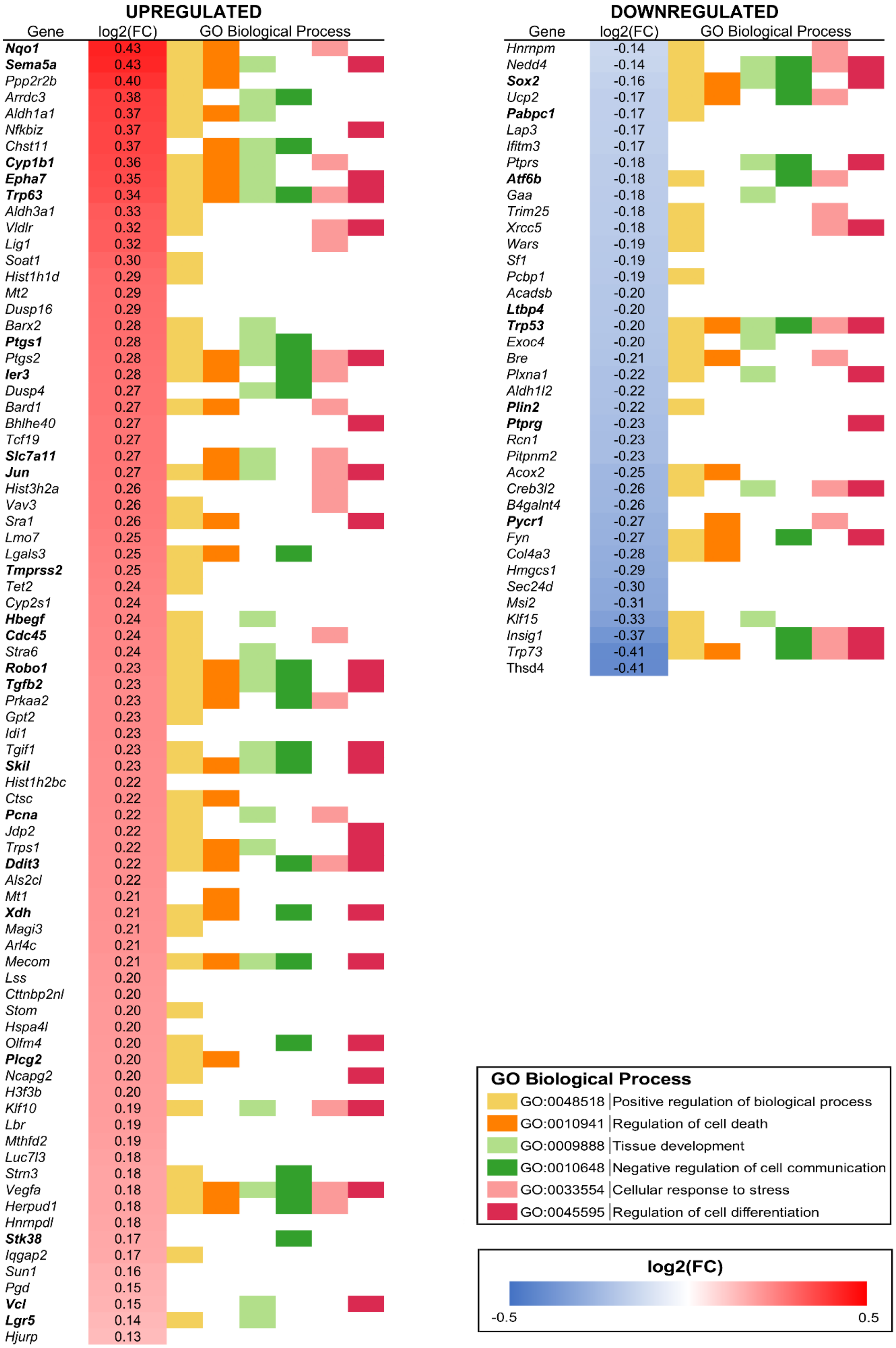
UPREGULATED
Gene
log2(FC)
GO Biological Process
Nqo1 0.43
Sema5a 0.43
Ppp2r2b 0.40
Arrdc3 0.38
Aldh1a1 0.37
Nfkbiz 0.37
Chst11 0.37
Cyp1b1 0.36
Epha7 0.35
Trp63 0.34
Aldh3a1 0.33
Vldlr 0.32
Lig1 0.32
Soat1 0.30
Hist1h1d 0.29
Mt2 0.29
Dusp16 0.29
Barx2 0.28
Ptgs1 0.28
Ptgs2 0.28
Ier3 0.28
Dusp4 0.27
Bard1 0.27
Bhlhe40 0.27
Tcf19 0.27
Slc7a11 0.27
Jun 0.27
Hist3h2a 0.26
Vav3 0.26
Sra1 0.26
Lmo7 0.25
Lgals3 0.25
Tmprss2 0.25
Tet2 0.24
Cyp2s1 0.24
Hbegf 0.24
Cdc45 0.24
Stra6 0.24
Robo1 0.23
Tgfb2 0.23
Prkaa2 0.23
Gpt2 0.23
Idi1 0.23
Tgif1 0.23
Skil 0.23
Hist1h2bc 0.22
Ctsc 0.22
Pcna 0.22
Jdp2 0.22
Trps1 0.22
Ddit3 0.22
Als2cl 0.22
Mt1 0.21
Xdh 0.21
Magi3 0.21
Arl4c 0.21
Mecom 0.21
Lss 0.20
Cttnbp2nl 0.20
Stom 0.20
Hspa4l 0.20
Olfm4 0.20
Plcg2 0.20
Ncapg2 0.20
H3f3b 0.20
Klf10 0.19
Lbr 0.19
Mthfd2 0.19
Luc7l3 0.18
Strn3 0.18
Vegfa 0.18
Herpud1 0.18
Hnrnpdl 0.18
Stk38 0.17
Iqgap2 0.17
Sun1 0.16
Pgd 0.15
Vcl 0.15
Lgr5 0.14
Hjurp 0.13
DOWNREGULATED
Gene
log2(FC)
GO Biological Process
Hnrnpm -0.14
Nedd4 -0.14
Sox2 -0.16
Ucp2 -0.17
Pabpc1 -0.17
Lap3 -0.17
Ifitm3 -0.17
Ptprs -0.18
Atf6b -0.18
Gaa -0.18
Trim25 -0.18
Xrcc5 -0.18
Wars -0.19
Sf1 -0.19
Pcbp1 -0.19
Acadsb -0.20
Ltbp4 -0.20
Trp53 -0.20
Exoc4 -0.20
Bre -0.21
Plxna1 -0.22
Aldh1l2 -0.22
Plin2 -0.22
Ptprg -0.23
Rcn1 -0.23
Pitpnm2 -0.23
Acox2 -0.25
Creb3l2 -0.26
B4galnt4 -0.26
Pycr1 -0.27
Fyn -0.27
Col4a3 -0.28
Hmgcs1 -0.29
Sec24d -0.30
Msi2 -0.31
Klf15 -0.33
Insig1 -0.37
Trp73 -0.41
Thsd4 -0.41
GO Biological Process
GO:0048518 Positive regulation of biological process
GO:0010941 Regulation of cell death
GO:0009888 Tissue development
GO:0010648 Negative regulation of cell communication
GO:0033554 Cellular response to stress
GO:0045595 Regulation of cell differentiation
log2(FC)
-0.5
0.5

Supplementary Figure S2: Influence of ablation of ten cells on airway organoids' transcriptome: list of differentially expressed genes that were up- (left) or downregulated (right) within 4.5 h after damage induction, and association to the top six enriched GO biological processes. Bold-written genes were discussed to be enriched in further mentioned GO biological processes. Data analysis was performed on the basis of RNA-seq data obtained from n = 6 samples per group.

2.2 Supplementary Videos

Supplementary Video S1: 3D projection of a multi-plane z-stack immunofluorescence image of an airway organoid stained for CK5 (red) and CK8 (green), as depicted in Figure S2. Individual underlying planes were captured with a distance of 2 µm each. CK5: cytokeratin 5; CK8: cytokeratin 8. Scale bar: 50 µm.

2.3 Supplementary Files

Supplementary File S1: Multi-plane z-stack immunofluorescence image of an airway organoid stained for CK5 (red) and CK8 (green). Individual planes were captured with a distance of 2 µm each. CK5: cytokeratin 5; CK8: cytokeratin 8. Scale bar: 50 µm.

Supplementary File S2: Multi-plane z-stack immunofluorescence image of an airway organoid stained for p63 (red) and CK8 (green). Individual planes were captured with a distance of 2 µm each. CK8: cytokeratin 8, p63: transformation related protein 63. Scale bar: 50 µm.

Supplementary File S3: Multi-plane z-stack immunofluorescence image of an airway organoid stained for p63 (red) and ACCTUB (cyan). Individual planes were captured with a distance of 2 µm each. ACCTUB: acetylated alpha-tubulin; 63: transformation related protein 63. Scale bar: 50 µm.
